# Phosphoethanolamine Transferase LptA in *Haemophilus ducreyi* Modifies Lipid A and Contributes to Human Defensin Resistance *In Vitro*

**DOI:** 10.1371/journal.pone.0124373

**Published:** 2015-04-22

**Authors:** Michael P. Trombley, Deborah M. B. Post, Sherri D. Rinker, Lorri M. Reinders, Kate R. Fortney, Beth W. Zwickl, Diane M. Janowicz, Fitsum M. Baye, Barry P. Katz, Stanley M. Spinola, Margaret E. Bauer

**Affiliations:** 1 Department of Microbiology and Immunology, Indiana University School of Medicine, Indianapolis, IN, United States of America; 2 Buck Institute for Research on Aging, Novato, CA, United States of America; 3 Department of Medicine, Indiana University School of Medicine, Indianapolis, IN, United States of America; 4 Department of Biostatistics, Indiana University School of Medicine, Indianapolis, IN, United States of America; 5 Department of Biostatistics, Richard M. Fairbanks School of Public Health, Indianapolis, IN, United States of America; 6 Department of Pathology and Laboratory Medicine, Indiana University School of Medicine, Indianapolis, IN, United States of America; 7 Center for Immunobiology, Indiana University School of Medicine, Indianapolis, IN, United States of America; University of Illinois at Urbana-Champaign, UNITED STATES

## Abstract

*Haemophilus ducreyi* resists the cytotoxic effects of human antimicrobial peptides (APs), including α-defensins, β-defensins, and the cathelicidin LL-37. Resistance to LL-37, mediated by the sensitive to antimicrobial peptide (Sap) transporter, is required for *H*. *ducreyi* virulence in humans. Cationic APs are attracted to the negatively charged bacterial cell surface. In other gram-negative bacteria, modification of lipopolysaccharide or lipooligosaccharide (LOS) by the addition of positively charged moieties, such as phosphoethanolamine (PEA), confers AP resistance by means of electrostatic repulsion. *H*. *ducreyi* LOS has PEA modifications at two sites, and we identified three genes (*lptA*, *ptdA*, and *ptdB*) in *H*. *ducreyi* with homology to a family of bacterial PEA transferases. We generated non-polar, unmarked mutants with deletions in one, two, or all three putative PEA transferase genes. The triple mutant was significantly more susceptible to both α- and β-defensins; complementation of all three genes restored parental levels of AP resistance. Deletion of all three PEA transferase genes also resulted in a significant increase in the negativity of the mutant cell surface. Mass spectrometric analysis revealed that LptA was required for PEA modification of lipid A; PtdA and PtdB did not affect PEA modification of LOS. In human inoculation experiments, the triple mutant was as virulent as its parent strain. While this is the first identified mechanism of resistance to α-defensins in *H*. *ducreyi*, our in vivo data suggest that resistance to cathelicidin LL-37 may be more important than defensin resistance to *H*. *ducreyi* pathogenesis.

## Introduction

*Haemophilus ducreyi*, the causative agent of the sexually transmitted disease chancroid, is a gram-negative obligate human pathogen [1,2]. Chancroidal infection has been shown to facilitate the transmission and acquisition of HIV [3]. Recent studies have demonstrated that *H*. *ducreyi* is also a prominent cause of non-sexually transmitted chronic cutaneous limb ulcerations in the South Pacific [4–6]. During infection, *H*. *ducreyi* encounters host immune cells and resident keratinocytes that secrete cationic antimicrobial peptides (APs), which target the bacterial cell membrane and lyse the cell [7–10]. *H*. *ducreyi* has been shown to resist the lethal activity of several classes of human APs, including α-defensins, β-defensins, and the human cathelicidin, LL-37 [11]. We previously identified two mechanisms of AP resistance in *H*. *ducreyi*, including the sensitive to antimicrobial peptides (Sap) transporter and the multiple transferable resistance (MTR) efflux pump. The Sap transporter confers resistance to LL-37 in vitro and is required for virulence in humans [12,13]. The MTR efflux pump confers resistance to LL-37 and to β-defensins in vitro [14]; its contribution to *H*. *ducreyi* pathogenesis in vivo has not yet been studied. Neither the Sap nor MTR transporter confers resistance to α-defensins [12–14].

One mechanism of AP resistance is to modify the cell surface with positively charged moieties, which results in electrostatic repulsion of the positively charged APs [15]. Frequently, gram-negative bacteria modify their lipopolysaccharide (LPS) or lipooligosaccharide (LOS) with positively charged aminoarabinose or phosphoethanolamine (PEA) [16–23]. *H*. *ducreyi* does not modify its LOS with aminoarabinose; however, *H*. *ducreyi* LOS contains one PEA on the lipid A and a second PEA on the KDO of its core oligosaccharide [24,25].

Modification of LPS or LOS with PEA has been shown to be advantageous for pathogenesis in several bacteria. In the pathogenic *Neisseria*, three PEA transferases have been identified, including LptA, Lpt-3 and Lpt-6, which modify the lipid A or the Heptose II core sugar at the third or sixth position, respectively. In *N*. *gonorrhoeae* and *N*. *meningitidis*, these PEA modifications contribute to resistance to polymyxin B, protegrin-1, and LL-37; resistance to human serum was conferred by PEA transferases in *N*. *gonorrhoeae* but not *N*. *meningitidis* [19,20,22,23]. PEA modification of lipid A in *N*. *gonorrhoeae* also contributes to survival in both the murine female genital tract and the human male urethra [26,27]. In *Salmonella enterica* and *Escherichia coli*, the PEA transferases CptA and EptB modify the Heptose II or KDO core sugars of the LPS, respectively; lipid A is also modified with PEA by EptA in *S*. *enterica* or PmrC in *E*. *coli* [18,28]. These PEA modifications of the LPS are important for resistance to polymyxin B in these enteric organisms [17,18,21]. Additionally, competitive infection experiments in mouse models of *S*. *enterica* infection showed a decrease in survival of PEA transferase mutants when compared to wild-type strains [17].

In addition to LPS and LOS, PEA transferases can modify other bacterial cell surface structures. Recently, studies have shown that the *Campylobacter jejuni* PEA transferase EptC modifies both lipid A and the flagellum with PEA; these modifications contribute to resistance of human and avian *β-*defensins and polymyxin B [29,30]. *N*. *gonorrhoeae* modifies its Type IV pili with PEA, although the function of this modification is unclear [31]. In addition to cell surface structures, the recently described PEA transferase OpgE (also known as YbiP) modifies osmoregulated periplasmic glucans (OPGs) in *E*. *coli* [32–34].

All characterized PEA transferases in gram-negative pathogens are members of the YhjW/YjdB/YijP/YbiP family of enzymatic inner membrane proteins [19]. The *H*. *ducreyi* genome encodes three genes that have strong homology to this family of PEA transferases (Table 1). We predicted that these genes, *lptA*, *ptdA*, and *ptdB*, contribute to AP resistance and virulence of *H*. *ducreyi*. In this study, we generated deletion mutants lacking one, two, or three putative PEA transferase genes in *H*. *ducreyi*. Using these mutants, we examined the role that these putative PEA transferase genes play in *H*. *ducreyi* resistance to APs. We also analyzed the contributions of these gene products to cell surface charge and LOS structure. Lastly, we utilized the human model of *H*. *ducreyi* infection to determine whether these PEA transferase genes are required for virulence in vivo.

**Table 1:** Putative *H*. *ducreyi* PEA transferases.

*H*. *ducreyi* Gene ID	*H*. *ducreyi* Protein	Homologous Protein (organism)	Expect Value	Site of Action
HD0852	Lipid A PEA transferase LptA	LptA (*N*. *gonorrhoeae*)	0	lipid A
		LptA (*N*. *meningitidis*)	0	lipid A
		EptA/PmrC (*E*. *coli*)	3e^-138^	lipid A
		EptA/PmrC (*S*. *enterica*)	2e^-134^	lipid A
HD0371	PEA transferase of *H*. *d* *ucreyi* PtdA	OpgE/YbiP (*E*. *coli*)	1e^-76^	unknown
HD1598	PEA transferase of *H*. *d* *ucreyi* PtdB	OpgE/YbiP (*E*. *coli*)	1e^-62^	unknown

## Materials and Methods

### Bacterial strains and growth conditions

Bacterial strains and plasmids are listed in Table 2. Unless otherwise mentioned, *H*. *ducreyi* strains were grown at 33°C with 5% CO_2_ on chocolate agar plates supplemented with 1% IsoVitalex. If strains contained plasmid vectors or antibiotic resistance cassettes, appropriate antibiotics were added to the agar, including spectinomycin (200 μg/ml), kanamycin (20 μg/ml) or streptomycin (100 μg/ml) [12]. Liquid cultures of *H*. *ducreyi* were grown in Columbia broth supplemented with hemin (50 μg/ml) (Aldrich Chemical Co., Milwaukee, WI), 5% heat inactivated fetal bovine serum (HyClone, Logan, UT), and 1% IsoVitalex and, when applicable, with half the concentration of appropriate antibiotics used in agar medium. For the human inoculation experiments, the bacteria were grown in a proteose-peptone based broth, as described [35]. *E*. *coli* strains were grown at 37°C in Luria-Bertani broth with appropriate antibiotics, which include spectinomycin (50 μg/ml), ampicillin (50 μg/ml), kanamycin (50 μg/ml), or streptomycin (100 μg/ml), with the exception of strain DY380, which was grown in L-Broth at 32°C or 42°C as indicated [36,37].

**Table 2:** Bacterial strains and plasmids used in study.

Strain or Plasmid	Genotype or Description	Source
**Strains**		
35000HP	Human-passaged variant of Class I clinical isolate 35000	[38]
35000HP/pLSSK	35000HP with vector pLSSK; StrepR^a^	[14]
*E*. *coli* TOP10	*F- mcrA Δ(mrr-hsdRMS-mcrBC) φ80lacZΔM15 ΔlacX74 recA1 araΔ139 Δ(ara-leu)7697 galU galK rpsL (StrR* ^*R*^ *) endA1 nupGλ-*	Invitrogen
*E*. *coli* DY380	*F- mcrA Δ(mrr-hsdRMS-mcrBC) Φ80dlacZ M15 ΔlacX74 deoR recA1 endA1 araD139 Δ(ara*, *leu) 7649 galU galK rspL nupG (λcI857 (cro-bioA) <> tet)*	[37]
35000HP*ΔptdA*	Unmarked *ptdA* deletion mutant of 35000HP	This study
35000HP*ΔlptA*	Unmarked *lptA* deletion mutant of 35000HP	This study
35000HP*ΔptdB*	Unmarked *ptdB* deletion mutant of 35000HP	This study
35000HP*ΔptdA lptA*	Unmarked *ptdA lptA* deletion mutant of 35000HP	This study
35000HP*ΔlptA ptdB*	Unmarked *lptA ptdB* deletion mutant of 35000HP	This study
35000HP*ΔptdA ptdB*	Unmarked *ptdA ptdB* deletion mutant of 35000HP	This study
35000HPΔPEAT	Unmarked *ptdA lptA ptdB* deletion mutant of 35000HP	This study
35000HPΔPEAT/pLSSK	35000HPΔPEAT with vector pLSSK; StrepR	This study
35000HPΔPEAT/pPEAT	35000HPΔPEAT with complement vector pPEAT; StrepR	This study
FX517	*dsrA*:*cat* insertion mutant of 35000; CmR	[39]
**Plasmids**		
pLSSK	*H*. *ducreyi* shuttle vector; StrepR	[40]
pPEAT	*ptdA lptA ptdB* in pLSSK; StrepR	This study
pRSM2072	Suicide vector; AmpR	[41]
pRSM2975	FLP recombinase vector	[36]
pMEB252	*ptdA* replaced with SpecR cassette in pRSM2072	This study
pMEB256	*lptA* replaced with SpecR cassette in pRSM2072	This study
pMEB251	*ptdB* replaced with SpecR cassette in pRSM2072	This study
pCR-XL-TOPO	TA cloning vector; KanR	Invitrogen
pMEB346	*ptdA* in pCR-XL-TOPO; KanR	This study
pMEB344	*lptA* in pCR-XL-TOPO; KanR	This study
pMEB348	*ptdB* in pCR-XL-TOPO; KanR	This study
pMEB355	*ptdA* in pLSSK; StrepR	This study
pMEB356	*lptA ptdB* in pCR-XL-TOPO; KanR	This study

^a^ StrepR, resistance to streptomycin; Cm^R^, resistance to chloramphenicol; AmpR, resistance to ampicillin; KanR, resistance to kanamycin; SpecR, resistance to spectinomycin.

### Deletion of *ptdA*, *lptA*, and *ptdB* in *H*. *ducreyi*

We used the recombineering technique, as described in [14], to generate unmarked, non-polar mutants with deletions in *lptA*, *ptdA*, or *ptdB*, as well as mutants with deletions in two or three of these genes (Table 2). This method allows for the deletion of the desired genes, leaving a short open reading frame that includes the start codon of the deleted gene, 27 codons encoding a FLP scar peptide, and the last 7 codons of the deleted gene [36]. Briefly, PCR products of 5.5 kb containing *ptdA*, 3.5 kb containing *lptA*, or 5.0 kb containing *ptdB* were generated with HD0371for3 and HD0371rev3, HD0852for1 and HD0852rev1, or HD1598for3 and HD1598rev3, respectively (Table 3). These products were cloned into *E*. *coli*, which was then transformed with a 2.2 kb PCR fragment containing a spectinomycin resistance (SpecR) cassette flanked by flippase (FLP) recognition target (FRT) sites and 50 bp of each respective target gene, which was amplified by H1P1HD0371 and H2P2HD0371, H1P1HD0852 and H2P2HD0852, or H1P1HD1598 and H2P2HD1598 (Table 3). After recombination, each fragment was ligated into pRSM2072 to generate the mutagenic plasmid pMEB252 (for *ptdA*), pMEB256 (for *lptA*), or pMEB251 (for *ptdB*).

**Table 3:** Primers used in this study.

Primer	Construct or use	Sequence
HD0371for3	*ptdA* fragment	ACTAGTGGCTCACCAAGCCATTGGTTACAA
HD0371rev3	*ptdA* fragment	ACTAGTGCAGGAATTGTACGGTCTGAACG
HD0852for1	*lptA* fragment	ACTAGTAGGGAAATGATCCGAAGCGAGGA
HD0852rev1	*lptA* fragment	ACTAGTTCGGTCGTATTAACGTGCTGACCA
HD1598for3	*ptdB* fragment	ACTAGTTGGCAAATTAAACCACACGCGGTC
HD1598rev3	*ptdB* fragment	ACTAGTATGCGCGATATGCTTAATGCTGGC
H1P1HD0371	pMEB252	AACAATGAGGCTATTTTATTTCTGCTGACCTTGTTTTATAGATTATTATGATTCCGGGGATCCGTCGACC
H2P2HD0371	pMEB252	ATTAACACATAGTTATTAATGCTTTCTAATTAATTGCTGATTGTGGTGTTTGTAGGCTGGAGCTGCTTCG
H1P1HD0852	pMEB256	ATAGCTTGATAGGCATTATTGCTTATGTTTTTATACAAAGGAATTTTATGATTCCGGGGATCCGTCGACC
H2P2HD0852	pMEB256	TTTTGCTAAAAAGGCCGCTTACAAGCGTATTACTCTACTTTATGAGCACATGTAGGCTGGAGCTGCTTCG
H1P1HD1598	pMEB251	ATTAAACAAGGAATAGCGCCCCTATATATTTACTACTAGAATCTATAATGATTCCGGGGATCCGTCGACC
H2P2HD1598	pMEB251	GCCTGCTTTTTTATTATTAGTAATCTGTGCTATTCCTGAACGTGCCCATTTGTAGGCTGGAGCTGCTTCG
HD0371compfor1	pMEB346	GCACGTGATGTATTGGCTAAAGGT
HD0371comprev3	pMEB346	TTTATTTTCTGCTTGCATTAACACATAGTTATTAAT
HD0852compfor1	pMEB344	GCTTGTAAAGGACGTAGCCAAGTG
HD0852comprev1	pMEB344	TACCGGCTCGGATTTCTAAGAAGG
HD1598compfor1	pMEB348	CCTTGCAATGCCTCACCACTTAGTT
HD1598comprev1	pMEB348	TCCGTCTCAATCAGTCGGTGACTA
HD0852for3	internal *lptA* probe	TTGTTTGTTCTCGTCGCAAACCCG
HD0852rev3	internal *lptA* probe	AGTAAGTTGGTCCATGGCTACCGA
HD0371for2	internal *ptdA* probe	ATCAGGAGAAGCAGGAGTTACTGG
HD0371rev2	internal *ptdA* probe	GTTGCCGGCGCACTAGCAATATAA
HD1598for2	internal *ptdB* probe	TGACGTTCTTGGTCGTCACTCGAA
HD1598rev2	internal *ptdB* probe	CCCGCCAAATACCACGATATCAAC

Each mutagenic plasmid was individually transformed into *H*. *ducreyi* as previously described [14], and mutant colonies were selected and transformed with pRSM2975, a temperature sensitive plasmid containing FLP recombinase. Incubation with anhydrous tetracycline induced the FLP recombinase, and after selection for loss of SpecR and loss of pRSM2975, the resulting recombined strain is an unmarked, non-polar deletion mutant lacking the target gene. This process was repeated with each mutagenic plasmid to create a strain collection lacking any combination of one, two, or three of the putative PEA transferase genes *ptdA*, *lptA*, and *ptdB* (Table 2). In all mutants, PCR and sequence analyses confirmed loss of target gene(s). For the mutant lacking all three genes, designated 35000HPΔPEAT (“PEAT” stands for “PEA Transferases”), whole genome sequencing revealed identical DNA between the mutant and parent strain 35000HP, with the exception of the loss of the three target genes. Growth curves indicated no significant changes in the growth rate of 35000HPΔPEAT compared to 35000HP, and outer membrane profiles showed no change in 35000HPΔPEAT when compared to 35000HP (data not shown). By silver-stained gel, the LOS banding pattern was identical between 35000HP and 35000HPΔPEAT (data not shown), which was expected, given the 123 Da molecular mass of PEA.

### Complementation of *ptdA*, *lptA*, and *ptdB* in trans

To complement the mutations in 35000HPΔPEAT, fragments of 2.05 kb, 2.5 kb, and 1.95 kb, containing 164 bp, 340 bp, and 185 bp 5’ of the *ptdA*, *lptA*, and *ptdB* ATG start sites, respectively, including the predicted promoter regions, were amplified from genomic DNA of 35000HP with primers HD0371compfor1 and HD0371comprev3, HD0852compfor1 and HD0852comprev1, and HD1598compfor1 and HD1598comprev1. These fragments were TA-cloned into pCR-XL-TOPO (Invitrogen, Carlsbad, CA), resulting in pMEB346 (containing *ptdA*), pMEB344 (containing *lptA*), and pMEB348 (containing *ptdB*).

The 2.05 kb *ptdA* fragment was excised from pMEB346 by digestion with NotI and SpeI and ligated into the shuttle vector pLSSK, resulting in the 5.6 kb plasmid pMEB355. The 2.5 kb *lptA* fragment of pMEB344 was excised by digestion with SpeI and XbaI and ligated into SpeI-digested, shrimp alkaline phosphatase-treated pMEB348 to form an 8.0 kb plasmid, pMEB356, which contained *lptA* followed by *ptdB* in the same orientation. The 4.5 kb *lptA ptdB* fragment was then excised from pMEB356 by digestion with SpeI and ApaI and ligated into pMEB355. The resulting plasmid, pPEAT, contains a 10.1 kb fragment containing *ptdA*, *lptA*, and *ptdB*, in the same orientation and along with their native promoter regions, in pLSSK. 35000HPΔPEAT was transformed with pPEAT to obtain 35000HPΔPEAT/pPEAT. Expression of *lptA*, *ptdA*, and *ptdB* in 35000HPΔPEAT/pPEAT was confirmed by qRT-PCR, as previously described [12], and the expression level of each PEA transferase gene was within ± 2-fold of parental expression levels (data not shown). To achieve isogenicity with the complemented mutant strain, all assays in which the parent, mutant, and complemented mutant strains were compared utilized pLSSK-bearing derivatives of the parent and mutant strains (35000HP/pLSSK and 35000HP*Δ*PEAT/pLSSK, respectively).

### Antimicrobial peptide bactericidal assays

Recombinant α- and β-defensin peptides were purchased from PeproTech Inc. (Rocky Hill, NJ: HNP-1, HBD-2, HBD-3), Peptides International (Louisville, KY: HD-5), and Sigma-Aldrich (St. Louis, MO: HNP-2); synthetic LL-37 was purchased from Phoenix Pharmaceuticals, Inc. (Belmont, CA). APs were reconstituted as previously described [14].

AP assays were performed as described previously [11]. Briefly, approximately 1000 CFU of mid-logarithmic phase *H*. *ducreyi* were incubated with 0.2, 2.0, and 20 μg/mL of diluted peptide for one hour. Samples were plated in triplicate on chocolate agar plates supplemented with appropriate antibiotics, and survival at each concentration of peptide after one hour was compared to survival of a control sample receiving diluent but no peptide. We used a Student’s t-test with Sidak adjustment for multiple comparisons to determine statistical significance, and comparisons between strains for sensitivity to a given AP were made only when assayed side-by-side.

### Serum bactericidal assay

35000HP/ pLSSK, 35000HPΔPEAT/pLSSK, and 35000HPΔPEAT /pPEAT were assayed for survival in human serum, as described previously [39]. Briefly, 14–16 hour growth from confluent plates was scraped into GC broth and diluted to 1000 CFU/ml. Bacteria were mixed 1:1 with either active or heat-inactivated human serum. Survival was determined by plate count after 45 min incubation. Assays were performed in triplicate, and the percent survival was calculated as the ratio of the (average active serum CFU/plate) / (average heat-inactivated serum CFU/plate) for each strain. The serum-sensitive, *dsrA*-deficient mutant strain FX517 was included in the assay as an internal control [39]; we used a Student’s t test with Sidak adjustment for multiple comparisons to determine statistical significance.

### Cell surface charge assay

We compared the cell surface charges of 35000HP, 35000HPΔPEAT, and 35000HPΔPEAT /pPEAT by adapting protocols which used the cationic dye Alcian Blue to compare membrane charge changes in red blood cells and yeast [42,43]. *H*. *ducreyi* strains were grown to mid-logarithmic phase, harvested, washed, and diluted in sterile phosphate buffered saline (PBS). Approximately 1000 CFU of bacteria were incubated for 30 minutes with 100 μg/mL Alcian Blue 8GX (Sigma-Aldrich, St. Louis, MO) in PBS; control samples were incubated in PBS alone. The bacteria were centrifuged and the supernatant removed, and the pellet was then suspended in 500 μL PBS. Absorbance at OD_607nm_ was measured for both the supernatant and suspended bacteria. Parallel bacterial samples were used to determine dry weight, and the absorbance measurements were normalized to the parent strain. Strains were compared for the amount of dye bound to the bacteria (by both loss of dye in supernatant and gain of dye in bacterial cells), normalized to the dry weight of each bacterial cell sample, using the PBS control samples to account for background absorption levels. A comparative decrease in remaining dye in the supernatant sample and a comparable increase in dye bound to the bacterial cells correlates with the bacterial cell having a more negative outer membrane charge.

For statistical analysis, mutant-parent pair differences in absorbance readings from both bacteria and supernatants from each strain were computed to account for day-to-day sample variation. Since the absorbance measurements were not normally distributed, non-parametric Wilcoxon signed rank tests were used to test for significant differences between mutant and parent samples.

### MALDI-MS analysis of *H*. *ducreyi* LOS

LOS was extracted from *H*. *ducreyi* using a microphenol method, as previously described [44]. Briefly, plate-grown bacteria were harvested, washed with PBS, and then diluted in H_2_O. The aqueous phase of a 65°C phenol/water extraction was kept, and the LOS was precipitated overnight at -20°C with cold ethanol and then lyophilized overnight. For each strain examined, LOS samples were prepared in triplicate.

To generate LOS amendable for mass spectrometric analyses, *O*-deacylated LOS (*O*-LOS) samples were prepared by treating 50–100 μg of LOS with 50 μl of anhydrous hydrazine followed by acetone precipitation as described previously [45]. All samples were desalted by drop dialysis using 0.025-μm pore size nitrocellulose membranes (Millipore, Bedford, MA) and were subsequently lyophilized. Samples were reconstituted in high-performance liquid chromatography (HPLC) grade H_2_O; 1 μl was loaded onto the target, allowed to dry, and then overlaid with either 1 μl of matrix (50 mg/ml 2,5-dihydroxybenzoic acid (DHB) (Laser Biolabs, Sophia-Antipolis Cedex, France) in 70% acetonitrile) or DHB made as a saturated solution in 70% acetonitrile. Samples were subsequently analyzed using matrix-assisted laser desorption ionization mass spectrometry (MALDI-MS) on an LTQ linear ion trap mass spectrometer coupled to a vMALDI ion source (MALDI-LIT) (Thermo Fisher, Waltham, MA). The vMALDI source uses a nitrogen laser that operates at 337.1-nm wavelength, 3-ns pulse duration, and 60-Hz repetition rate. Data were collected in the negative ion mode using the automated gain control and the automatic spectrum filter settings. Alternatively, samples were analyzed on the Waters Synapt G2 hybrid mass spectrometer utilizing the MALDI ionization source in the negative ion mode.

### Human model of *H*. *ducreyi* infection

Human subjects research was performed in accordance with the human experimentation guidelines of the U.S. Department of Health and Human Services and the Institutional Review Board of Indiana University–Purdue University Indianapolis under study #93–1237. This study was reviewed and approved by the Institutional Review Board of Indiana University-Purdue University-Indianapolis prior to initiation of the experiments involving human subjects. Prior to participation, written informed consent was obtained from each human subject for study participation and for HIV serology.

For the human model of *H*. *ducreyi* infection, we used the strains 35000HP and 35000HPΔPEAT. Three healthy male and five healthy female volunteers over 21 years of age were recruited for the study.

The experimental human challenge protocols were followed as previously described [35,38,46–49]. Subjects were inoculated on the upper arm; one arm was inoculated at 3 sites with a targeted dose of 90 CFU of the parent strain, and the other arm was inoculated at 3 sites with targeted doses of 45, 90, and 180 CFU of the mutant strain (see Results section for the actual inoculum doses achieved). Subjects were observed until they reached the clinical endpoint, defined as either 14 days post-inoculation, resolution of infection at all sites, or development of a pustule that was painful or pruritic or at least 6 mm in diameter. The clinician following the participants were blinded to the identity of the arm that had been inoculated with the mutant or parent strains. Following the clinical endpoint, the code was broken, and biopsies were taken of both a parent site and mutant site. The subjects were then treated with a single dose of oral ciprofloxacin. Papule and pustule formation rates for parent and mutant inoculation sites were compared using logistic regression with generalized estimating equations (GEE) to account for the within-subject correlation. Ninety-five percent confidence intervals (95% CI) for papule and pustule formation rates were calculated using GEE-based sandwich standard errors. Day 1 papule size was compared using analysis of variance with a random subject effect.

To confirm that the inocula contained the correct strains and that there was no cross contamination of the inoculated sites, DNA hybridization was performed on colonies derived from the inocula and from surface cultures and biopsies of infected sites. Probes specific for *dnaE*, *lptA*, *ptdA*, and *ptdB* were amplified using primers reported previously [36], and HD0852for3/HD0852rev3, HD0371for2/HD0371rev2, and HD1598for2/HD1598rev2, respectively (Table 3). The probes were labeled with digoxigenin using the DIG DNA labeling kit and detected using the DIG Easy Hyb protocol (Roche Applied Sciences) as described [36].

## Results

### Identification of putative PEA transferases in *H*. *ducreyi*

Previously, we established that the Sap transporter and MTR efflux pump mediated LL-37 and β-defensin resistance in *H*. *ducreyi* [12–14]. We wanted to identify a mechanism responsible for α-defensin resistance. Similar to other bacteria, *H*. *ducreyi* LOS is modified with the positively charged PEA, leading to our hypothesis that *H*. *ducreyi* PEA modification confers resistance to APs. Homology searches found three members of the YhjW/YjdB/YijP/YbiP family in the *H*. *ducreyi* genome (Table 1). *HD0852 (lptA)* shared strong homology with lipid A PEA transferase genes in *Neisseria*, *E*. *coli*, and *S*. *enterica*. *HD0371* (*ptdA)* and *HD1598* (*ptdB*) were homologous to the OPG-modifying PEA transferase OpgE in *E*. *coli*.

### The *H*. *ducreyi* PEA transferase genes confer resistance to α- and β-defensins but not to cathelicidin or human serum

In order to determine the contribution of each putative PEA transferase gene to AP resistance, mutants were made with deletions in one, two, or all three genes (Table 2). This panel of mutants was used to assess the role of these genes in resistance to human APs relevant to *H*. *ducreyi* infection, including APs secreted by resident keratinocytes (β-defensins, LL-37 and, in vaginal epithelial cells, α-defensin HD-5) or infiltrating neutrophils (α-defensins, HBD-4, and LL-37) and macrophages (β-defensins and LL-37) [7–9]. Initial assays used HD-5 and HBD-3 as representative α- and β-defensins, respectively, and the cathelicidin LL-37.

**Fig 1:**
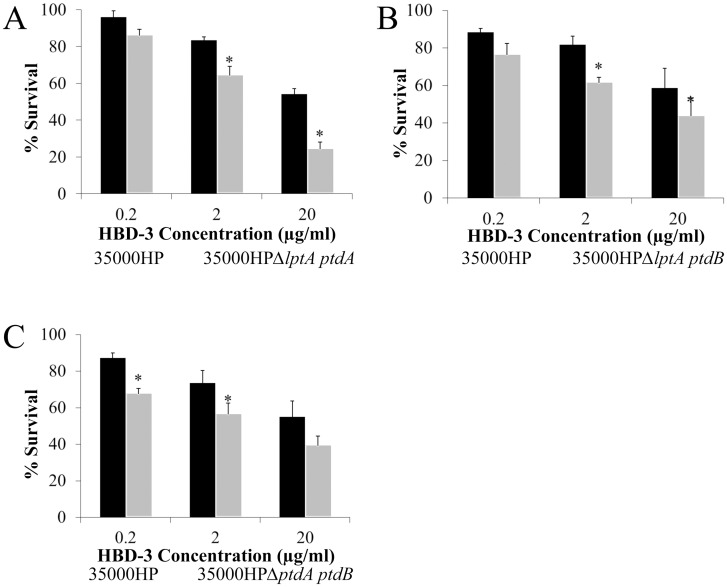
Deletion of two putative PEA transferase genes in *H*. *ducreyi* increases susceptibility to HBD-3. (A) 35000HP*ΔlptA ptdA*, (B) 35000HP*ΔlptA ptdB*, and (C) 35000HP*ΔptdA ptdB* were compared with 35000HP for resistance to the β-defensin HBD-3. All three mutants lacking two putative PEA transferase genes were significantly more sensitive than 35000HP to HBD-3, indicated by asterisks (*P* < 0.05). Data represent average ± standard error of 3–4 independent assays, and statistical significance was determined by Student’s t-test.

We found that loss of any one PEA transferase gene had no significant effect on AP resistance (data not shown). When two PEA transferase genes were deleted, all double mutant combinations were significantly more susceptible than 35000HP to the β-defensin HBD-3 at multiple concentrations (Fig 1). These mutants with deletions in two PEA transferase genes showed no increase in sensitivity to HD-5 or LL-37 (data not shown).

**Fig 2:**
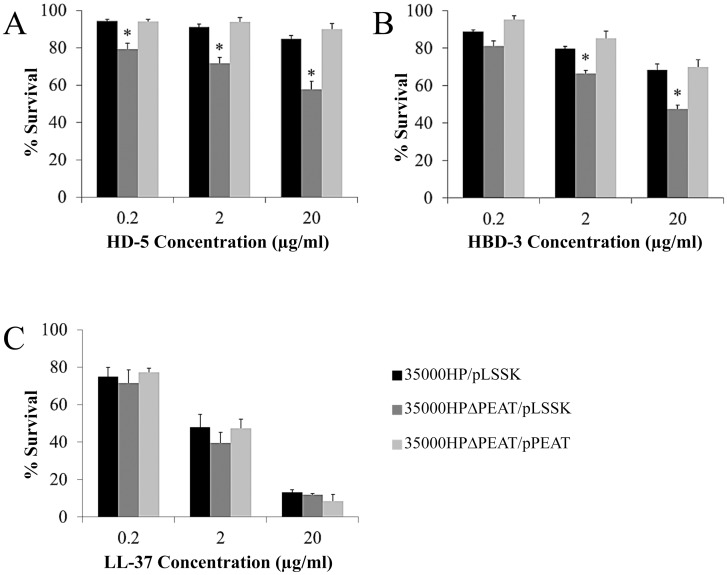
*H*. *ducreyi* PEA transferases confer resistance to α- and β-defensins. 35000HP, 35000HPΔPEAT and 35000HPΔPEAT/pPEAT were tested for resistance to the (A) α-defensin HD-5 (B) β-defensin HBD-3, and (C) human cathelicidin LL-37. Asterisks indicate statistically significant differences from 35000HP (*P* < 0.05). Complementation with pPEAT restored parental levels of susceptibility to defensins. Data represent average ± standard error of six independent replicates, and statistical significance was determined by Student’s t-test.

To determine the collective contribution of all three PEA transferase genes to AP resistance, we assayed 35000HP/pLSSK, 35000HPΔPEAT/pLSSK, and 35000HPΔPEAT/pPEAT for sensitivity to α-defensins, β-defensins, and LL-37 at various concentrations. We found that 35000HPΔPEAT was significantly more sensitive to the α-defensin HD-5 at all concentrations tested; complementation with pPEAT restored the parental resistance phenotype (Fig 2A). Similar results were obtained when the parent, mutant, and complemented mutant were challenged with the additional human α-defensins HNP-1 and HNP-2 (S1 Fig). These data indicate that the *lptA*, *ptdA*, and *ptdB* gene products contribute to α-defensin resistance in *H*. *ducreyi*.

Next, we found that 35000HPΔPEAT was significantly more susceptible to the β-defensin HBD-3 at 2 concentrations, and complementation with pPEAT restored the parental resistance phenotype (Fig 2B). Similar results were obtained when the parent, mutant, and complemented mutant were challenged with the human β-defensin HBD-2 (S1 Fig). These data indicate that the *lptA*, *ptdA*, and *ptdB* gene products contribute to β-defensin resistance in *H*. *ducreyi*.

We found no significant difference in sensitivity to LL-37 between 35000HP and 35000HPΔPEAT at any concentration (Fig 2C). Taken together, these data suggest that the *H*. *ducreyi* PEA transferase gene products likely contribute to α- and β-defensin resistance but do not contribute significantly to cathelicidin resistance.

**Fig 3:**
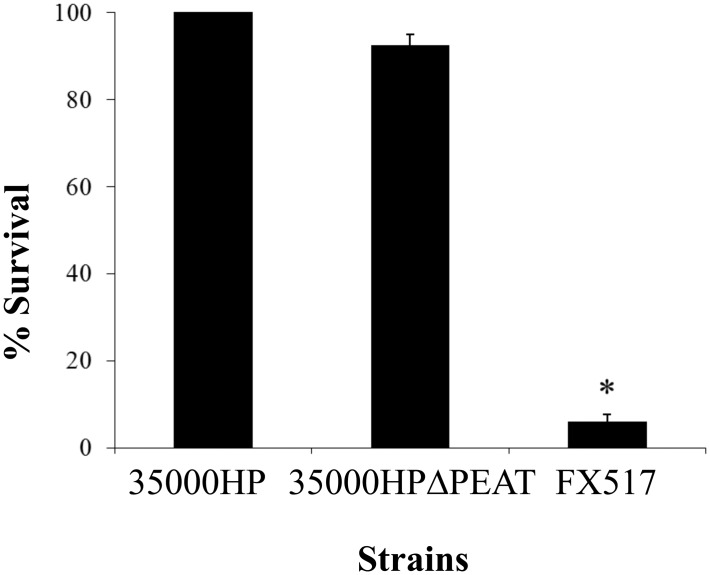
*H*. *ducreyi* PEA transferases do not confer resistance to human serum. 35000HP, 35000HPΔPEAT and FX517 were examined for resistance to human serum. There was no significant difference in sensitivity to serum between 35000HP and 35000HPΔPEAT; FX517, a Δ*dsrA* mutant that served as a control for serum sensitivity, was significantly more sensitive to serum than 35000HP, indicated by asterisk (*P* < 0.05). Data represent average ± standard error of six independent assays, and statistical significance was determined by Student’s t-test.

Addition of PEA to LOS contributes to serum resistance in *N*. *gonorrhoeae* [22]. In contrast, we found no significant difference in sensitivity to normal human serum between 35000HP and 35000HPΔPEAT (Fig 3). These data indicate that the putative PEA transferases of *H*. *ducreyi* likely do not contribute to resistance to human serum.

### 35000HPΔPEAT had a more negative cell surface charge than 35000HP

Once we established that the PEA transferase genes contributed to defensin resistance, we next examined whether these transferase genes affected the cell surface charge of *H*. *ducreyi*. To compare the relative cell surface charges of 35000HP and 35000HPΔPEAT, we modified a protocol that uses the cationic dye Alcian Blue 8GX, which proportionately binds to the cell surface based on charge.

When comparing 35000HPΔPEAT to 35000HP (Table 4), we found 17.5% more dye bound to 35000HPΔPEAT cells than to 35000HP cells (*P* < 0.0001). We also found 16.5% less dye remaining in the supernatant from 35000HPΔPEAT cells than from 35000HP cells (*P* < 0.0001). Complementation with the three PEA transferase genes restored the level of dye bound to that of 35000HP (data not shown). These data indicated that the cell surface of 35000HPΔPEAT was more negatively charged than 35000HP, suggesting that the putative PEA transferase gene products conferred the addition of positive moieties on the cell surface.

**Table 4:** Cell surface of 35000HPΔPEAT is more negatively charged than 35000HP.

Sample	Strain	Mean	S.D.^b^	Median	Wilcoxon p-value^a^
**Bacterial Cells**	35000HP	0.280	0.203	0.217	<0.0001
	35000HPΔPEAT	0.329	0.237	0.268	
**Supernatant**	35000HP	0.588	0.602	0.374	<0.0001
	35000HPΔPEAT	0.491	0.532	0.313	

^a^ Statistical significance determined by comparing median values with a non-parametric Wilcoxon signed ranks test because the absorbance measurements were not normally distributed; experimental n = 24

^b^ S.D., Standard deviation

### *H*. *ducreyi* LptA modifies lipid A with phosphoethanolamine.

After determining that the PEA transferase genes played a role in modifying the *H*. *ducreyi* cell surface charge, we hypothesized that the observed surface charge effects would correlate with PEA modification of *H*. *ducreyi* LOS. We tested this hypothesis by analyzing the LOS structures of each single mutant as well as the parent, triple mutant, and complemented triple mutant via mass spectrometry.

LOS samples from each strain were *O*-deacylated to generate water soluble *O*-LOS that was analyzed by MALDI-MS. Lipid A and oligosaccharide (OS) “prompt fragments” are generated within the instrument during the ionization process. These fragments can provide useful information in determining LOS structures, as they allow one to observe the masses of these individual components of the *O*-LOS in the same analyses as the intact *O*-LOS structures.

**Fig 4:**
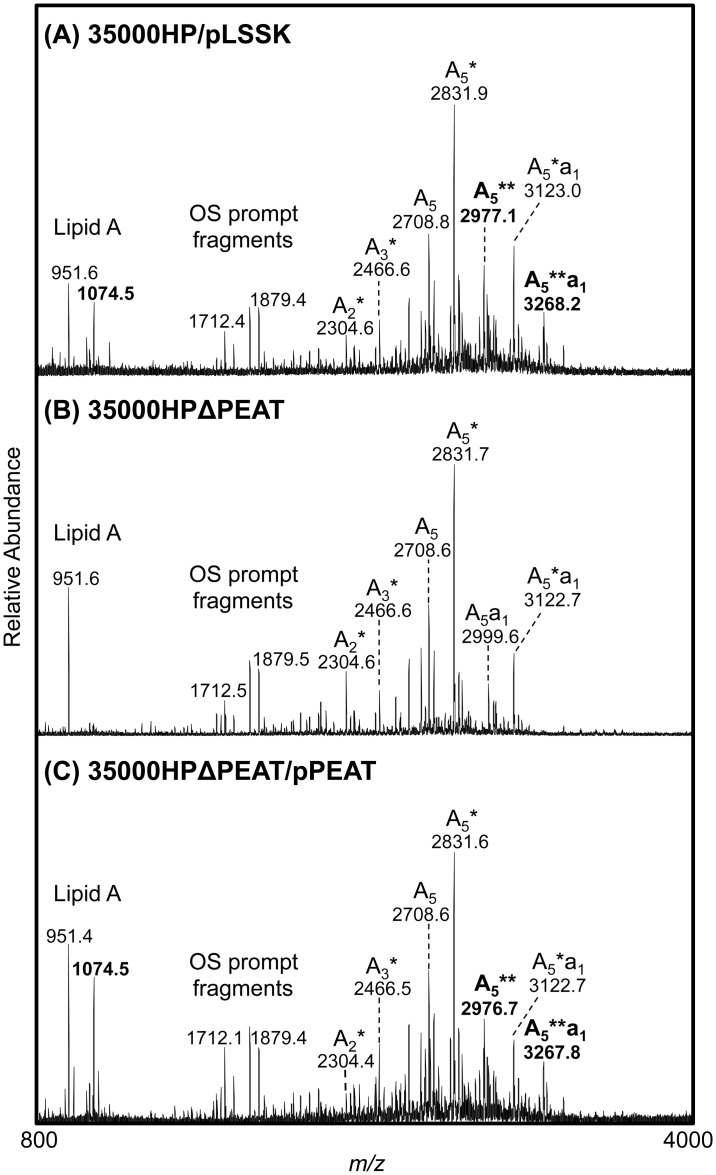
*H*. *ducreyi* putative PEA transferases contribute to modification of lipid A with PEA. Negative-ion MALDI-MS spectra of *O*-LOS from (A) 35000HP/pLSSK, (B) 35000HPΔPEAT/pLSSK, and (C) 35000HPΔPEAT/pPEAT. The compositions of the glycoforms are described in Table 5. Masses labeled in bold were only observed in the parent strain and the corresponding PEAT complemented strain. The asterisk in the glycoform nomenclature designates the number of PEA groups present on the *O*-LOS.

To define the full contribution of the putative PEA transferases to LOS structure, triplicate *O*-LOS samples from 35000HP/pLSSK, 35000HPΔPEAT/pLSSK, and 35000HPΔPEAT/pPEAT were prepared and analyzed by MALDI-MS. Fig 4 and Table 5 show representative data from these analyses. The *O*-deacylated monophosphorylated lipid A (MPLA) at *m/z* 951.6 or 951.4 was observed in 35000HP/pLSSK as well as in 35000HPΔPEAT and its corresponding complemented strain. The peak at *m/z* 1074.5, which corresponds to a MPLA with the addition of one PEA (+ 123 Da), was observed in the parent strain and the complemented mutant strain, but was not observed in 35000HPΔPEAT. Thus, 35000HPΔPEAT lacked the wild-type PEA modification of its lipid A.

**Table 5:** *H*. *ducreyi O*-LOS glycoforms and corresponding monoisotopic masses.

						[M-H]^-^_obs_		
Glycoform^*a*^	Hex	HexNAc	PEA	NeuAc	[M-H]^-^_calc_	35000HP/ pLSSK	35000HP ΔPEAT/ pLSSK	35000HP ΔPEAT/ pPEAT
^*b*^ **A**_**5**_ **a**_**1**_ **	3	1	2	1	**3268.07**	**3268.18**	^*c*^ **nd**	**3267.82**
A_5_a_1_*	3	1	1	1	3123.08	3123.00	3122.73	3122.73
A_5_a_1_	3	1	0	1	3000.07	2999.91	2999.73	2999.55
^*b*^ **A**_**5**_ **	3	1	2	0	**2976.98**	**2977.09**	**nd**	**2976.73**
A_5_*	3	1	1	0	2831.98	2831.91	2831.73	2831.64
A_5_	3	1	0	0	2708.98	2708.82	2708.64	2708.55
A_4_*	2	1	1	0	2669.93	2669.82	2669.64	2669.55
A_4_	2	1	0	0	2546.92	2546.73	2546.64	2546.55
A_3_*	2	0	1	0	2466.85	2466.64	2466.64	2466.45
A_3_	2	0	0	0	2343.84	2343.64	2343.55	2343.45
A_2_*	1	0	1	0	2304.80	2304.55	2304.55	2304.36

^a^ All molecular masses contain four Heptoses, Kdo(P), and *O*-deacylated lipid A. Asterisks indicate the number of PEA present in the structure.

^b^Glycoforms were observed in their sodiated forms.

^c^nd indicates that the particular glycoform was not detected in the sample.

Full *O*-LOS glycoforms, which contain *O*-deacylated lipid A, the core, and varying lengths of the branch oligosaccharide, are shown in Table 5 for each strain. Evaluation of these *O*-LOS glycoforms showed that all three strains had PEA present on the oligosaccharide. Glycoforms containing one PEA were observed in all three strains at *m/z* 3123, 2832, 2670, 2467, and 2304 (Table 5). Glycoforms containing two PEA groups were observed in both the parent strain and the complemented mutant strain at *m/z* 3268 and 2977 (Table 5); these data are consistent with previous work showing that *H*. *ducreyi* LOS contains one PEA on its lipid A and one PEA on its core oligosaccharide. In contrast, the masses corresponding to *O*-LOS structures with two PEA groups were not observed in 35000HPΔPEAT (Table 5). The lack of PEA on the lipid A from 35000HPΔPEAT, combined with the presence of one PEA on the oligosaccharide of the *O*-LOS from this strain, demonstrated that, in this mutant, the lipid A PEA transferase was inactive but a second PEA transferase, responsible for the addition of PEA onto the oligosaccharide, was still active.

**Fig 5:**
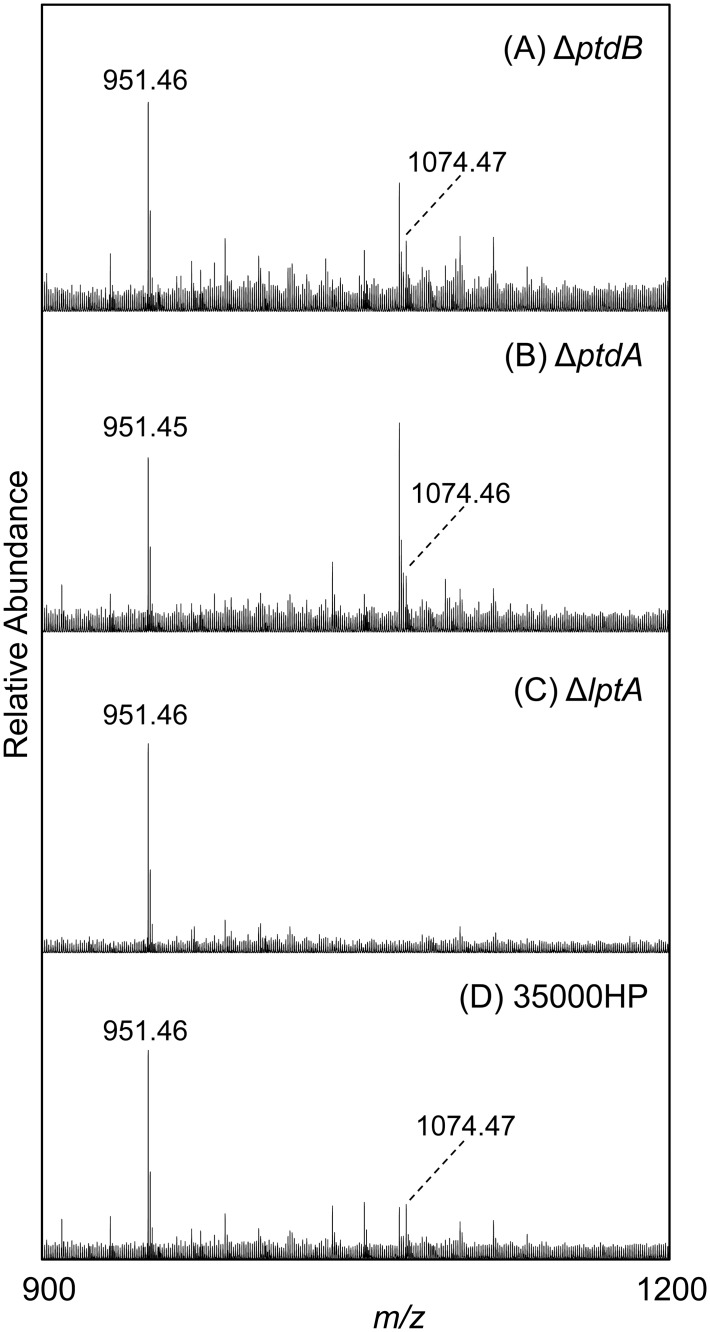
LptA contributes to modification of lipid A with PEA. Negative-ion MALDI-MS spectra of *O*-LOS from (A) 35000HP*ΔptdB*, (B) 35000HP*ΔptdA*, (C) 35000HP*ΔlptA*, and (D) 35000HP. The Fig shows zoomed images from representative spectra for each strain. The *O*-deacylated monophosphorylated lipid A (MPLA) was observed at *m/z* 951.46 or 951.45, this structure plus the addition of PEA was observed at *m/z* 1074.46 or 1074.47. The MPLA plus PEA was not observed in the 35000HP*ΔlptA* samples.

The lipid A prompt fragments provided the most useful information for the analyses of the *O*-LOS from the single mutants strains. Representative spectra of the lipid A regions of the *O*-LOS from the single mutant strains and the corresponding parent strain are shown in Fig 5. The peak at *m/z* 951.5, corresponding to the MPLA, was observed in all three single mutants (35000HP*ΔptdA*, 35000HP*ΔptdB*, and 35000HP*ΔlptA*) as well as the parent strain. The MPLA with the addition of one PEA, found at *m/z* 1074.5, was observed in the parent strain as well as the 35000HP*ΔptdA* and 35000HP*ΔptdB* strains, but was not observed in the 35000HP*ΔlptA* strain. These data indicate that LptA confers the PEA modification to lipid A.

### The *H*. *ducreyi lptA*, *ptdA*, and *ptdB* genes are not required for survival in vivo

We next examined the role of the PEA transferase genes in virulence in vivo by using the human model of *H*. *ducreyi* infection. To do this, we challenged eight healthy adult volunteers with 58–139 CFU of 35000HP and 40–224 CFU of 35000HPΔPEAT (Table 6).

**Table 6:** 35000HPΔPEAT is fully virulent in vivo.

Response to inoculation of live *H*. *ducreyi* strains						
Volunteer^a^	Gender^b^	Observation period (days)	Isolate^c^	Dose (CFU)	No. of initial papules	No. of pustules at endpoint
441	M	7	P	58	3	1
			M	56–224^d^	2	2
442	M	7	P	58	3	1
			M	56–224^d^	3	1
444	F	9	P	70	3	0
			M	40–159^e^	3	1
445	F	7	P	70	3	1
			M	40–159^e^	2	0
446	F	7	P	70	3	1
			M	40–159^e^	3	0
447	M	8	P	139	3	0
			M	112	3	0
451	F	7	P	108	3	0
			M	88	3	0
453	F	12	P	108	2	1
			M	88	2	1

^a^ Volunteers 441 and 442 were inoculated in the first iteration. Volunteers 444, 445, and 446 were inoculated in the second iteration. Volunteer 447 was inoculated in the third iteration. Volunteers 451 and 453 were inoculated in the fourth iteration.

^b^ M, Male; F, Female

^c^ P, 35000HP (parent); M, 35000HPΔPEAT (mutant)

^d^ Mutant-inoculated sites received estimated delivered doses of 56, 112, or 224 CFU.

^e^ Mutant-inoculated sites received estimated delivered doses of 40, 80, or 159 CFU.

Of sites inoculated with 35000HP, 23 of 24 (95.8%) developed papules, while 21 of 24 (87.5%) sites inoculated with 35000HPΔPEAT developed papules. The average parent papule size at day 1 was 8.7 mm^2^ whereas the average mutant papule size at day 1 was 5.0 mm^2^. Pustules developed at 5/24 (20.8%) locations for both the 35000HP and 35000HPΔPEAT inoculated sites.

Statistical comparisons between 35000HP and 35000HPΔPEAT showed no difference between their papule formation rates (parent = 95.8% [95% C.I., 88.2–99.9%], mutant = 87.5% [95% C.I., 76.3–98.7%], *P* = 0.103) and their pustule formation rates (parent = 20.8% [95% C.I., 9.7–32.0%], mutant = 20.8% [95% C.I., 4.8–36.9%], *P* = 1.0). The difference in day 1 papule size (parent 8.7 mm^2^, mutant 5.0 mm^2^) did approach statistical significance (*P* = 0.051). Taken together, these data suggest that 35000HPΔPEAT does not contribute significantly to *H*. *ducreyi* virulence during experimental human infection.

For each volunteer that developed pustules at both parent and mutant-inoculated sites, one parent site and one mutant site were biopsied, stained with hematoxylin-eosin and anti-CD3 antibodies, and semi-quantitatively cultured as described [47]. All samples contained pustules that eroded through the epidermis and a dense dermal infiltrate that predominantly consisted of T cells (data not shown); mutant and parent sites were histopathologically indistinguishable.

All colonies recovered from the inocula, surface cultures and biopsies were tested for the presence of *dnaE*, *lptA*, *ptdA*, and *ptdB* sequences by colony hybridization. The *dnaE* probe hybridized to all the colonies tested from both parent (N = 141) and mutant (N = 142) inocula, while the *lptA*, *ptdA*, and *ptdB* probes only hybridized to colonies from the parent inocula. At least one positive surface culture for *H*. *ducreyi* was obtained during follow-up visits from 8% of the parent sites and 8% of the mutant sites. The *dnaE* probe hybridized to colonies from both parent (N = 94) and mutant (N = 70) inoculated sites, while the *lptA*, *ptdA*, and *ptdB* probes only bound to colonies from the parent sites. All 4 paired biopsies of mutant and parent pustules yielded *H*. *ducreyi*. The *dnaE* probe hybridized to all of both parent (n = 103) and mutant (n = 107) colonies obtained from the biopsies, while the *lptA*, *ptdA*, and *ptdB* probes only hybridized to colonies obtained from the parent biopsies. Thus, all colonies derived from the inocula and the infected sites had the expected genotypes.

## Discussion

In gram-negative bacteria, PEA transferases modify a variety of substrates, including carbohydrate components of LPS, other sugars in the cell wall, and proteinaceous surface structures; the best characterized PEA transferases are those that modify the LPS of enteric pathogens or the LOS of *N*. *gonorrhoeae*. PEA modification contributes to AP resistance and serum resistance in vitro and to virulence in vivo. In *H*. *ducreyi*, both lipid A and core components of the LOS are modified with PEA. In this study, we examined three *H*. *ducreyi* genes with strong homology to known PEA transferases.

We examined the effect of PEA modification on resistance to human APs relevant to *H*. *ducreyi* infection, including α-defensins, β-defensins, and the cathelicidin LL-37. PEA modification did not appear to confer resistance to LL-37 (Fig 2C); however, previous studies have already established that both Sap and MTR play major roles in LL-37 resistance [12–14]. The presence of an intact Sap transporter and a functioning MTR efflux pump could mask any contribution of PEA modification to LL-37 resistance. Clearly, however, PEA modification plays a role in *H*. *ducreyi* resistance to α- and β-defensins. When any one putative PEA transferase gene was deleted, we observed no difference in AP susceptibility between the parent and mutant strains; however, when two of these genes were deleted, the mutants became more susceptible to β-defensins (Fig 1). When all three putative PEA transferase genes were deleted, the mutant became more susceptible than the parent strain to α-defensins. Thus, all three putative PEA transferases contribute to defensin resistance.

When all three putative PEA transferases were deleted, we also observed a significant change in surface charge of the organism (Table 4). Loss of only one putative PEA transferase gene had no effect on surface charge in our assay (S2 Fig), which may reflect a lack of sensitivity of the cationic dye assay. We also detected an increase in negativity in the cell surface in 35000HP*ΔptdA ptdB* (S2 Fig), which correlates with its increased sensitivity to β-defensins; these data indicate that PtdA and PtdB affect cell surface charge independent of LptA. However, the greatest effect on surface charge was observed in the triple mutant and correlated with its increased sensitivity to both α- and β-defensins. Thus, the additive effects of these three gene products are what provides sufficient cell surface positivity to repel cationic APs.

In contrast to observations in *N*. *gonorrhoeae*, loss of the three putative PEA transferase genes did not affect the susceptibility of *H*. *ducreyi* to human serum [22]. Our results indicate that in *H*. *ducreyi*, as in *N*. *meningitidis* [17], PEA modification likely does not contribute to complement evasion. *H*. *ducreyi* expresses three surface proteins, the *ducreyi* serum resistance protein DsrA, the *ducreyi* lectin DltA, and the major outer membrane protein MOMP, that have been previously shown to confer resistance to complement-mediated killing [39,50,51]. DsrA blocks binding of IgM to the *H*. *ducreyi* surface and prevents initiation of the complement cascade, whereas the contributions of DltA and MOMP to serum resistance remain unclear [39,50]. Thus, in *H*. *ducreyi*, the activities of DsrA and other outer membrane proteins may be masking any contribution of PEA modification on serum resistance.

As discussed above, our data suggest that all three putative PEA transferase genes in *H*. *ducreyi* contribute to lessening the negativity of the cell surface (Table 4). Mass spectrometric analysis indicated that LptA functions by modifying lipid A with PEA (Figs 4–5); the functions of PtdA and PtdB are less clear. Loss of either gene product had no effect on the modification of lipid A (Fig 5), but both gene products contributed to AP resistance (Figs 1–2). These genes also contributed to effects on cell surface charge even in the absence of LptA (S2 Fig). The closest homolog of these genes, *opgE*, encodes a PEA transferase that targets OPGs. OPGs and related periplasmic glucans have been found in disparate gram-negative bacteria [32]. However, no OPG-like molecules and no OPG biosynthetic genes, except for *opgE*, have been found among the *Pasteurellaceae*, which includes *H*. *ducreyi*. Thus, OpgE homologs in the *Pasteurellaceae*, such as PtdA and PtdB, likely target other molecules; our data suggest that PtdA and PtdB affect other surface structures. There is precedence for PEA transferases to modify surface proteins. *C*. *jejuni* modifies its flagellum with PEA, and *N*. *gonorrhoeae* modifies its type IV pilus with PEA [29,31]. *H*. *ducreyi* does not produce these surface structures; we are currently investigating the role that PtdA and PtdB play in modification of surface components other than LOS.

The other question raised by the mass spectrometric analyses is what gene product is responsible for modifying the *H*. *ducreyi* KDO with PEA? The PEA modification of KDO was detected by mass spectrometry in all strains examined in this study, including the triple mutant (Fig 4). These data indicate that an additional PEA transferase exists in *H*. *ducreyi* that has yet to be identified. Studies are currently underway to find this transferase and to determine how the LOS core KDO is modified with PEA.

When tested in the human model of *H*. *ducreyi* infection, 35000HPΔPEAT was fully virulent. This result differs from previous in vivo studies in animal and human models of *S*. *enterica* and *N*. *gonorrhoeae* infection. An *eptA cptA* mutant of *S*. *enterica* was modestly less fit than its parent strain in mice [17]. In *N*. *gonorrhoeae*, LptA-mediated PEA modification of lipid A provided a significant fitness advantage in both female mice and human male volunteers [26].

In the *H*. *ducreyi* human challenge model, the initial disease (papules) at inoculated sites may spontaneously resolve or progress to pustule formation; the overall parent pustule formation rate is 53.8% (n = 803 sites). In the present study, the pustule formation rate at parent-inoculated sites was only 20.8%. With such a low parent pustule formation rate, it would be difficult to discern a difference in virulence between parent and mutant strains even if the mutant was attenuated. It is also possible that the difference in susceptibility to defensins between the parent and mutant strains observed in vitro may not be significant enough to correlate with attenuation in vivo. Since 35000HPΔPEAT still has a PEA modification on its LOS, we cannot say for certain that PEA transferases are not necessary for virulence in *H*. *ducreyi*; however, these data show that *lptA*, *ptdA*, and *ptdB* are not required for *H*. *ducreyi* virulence in the human model of infection.

The results of this trial raise the question of the importance of defensins in the host’s response to *H*. *ducreyi* infection. Previous human trials examining the importance to pathogenesis of the Sap transporter, which confers resistance to LL-37 but not defensins, established a direct correlation between LL-37 resistance in vitro and virulence in vivo: a *sapA* mutant with partial loss of transporter-mediated LL-37 resistance was partially attenuated in vivo, and a *sapBC* mutant, with no transporter activity, was fully attenuated for virulence [12,13]. Taken together, these studies suggest that, during *H*. *ducreyi* infection, LL-37 plays a more significant role than defensins in host defense. A greater impact of LL-37 than defensins on host defense could also account for the greater role observed for *N*. *gonorrhoeae* LptA in human infection; unlike its *H*. *ducreyi* homolog, *N*. *gonorrhoeae* LptA contributes to LL-37 resistance [22].

In summary, we identified three *H*. *ducreyi* genes that shared a high degree of homology to known PEA transferases in other gram-negative bacteria. Deletion of all three genes resulted in greater sensitivity to α- and β-defensins, a more negatively charged cell surface, and the loss of PEA modification on lipid A. However, the triple mutant was fully virulent when tested in the human model of *H*. *ducreyi* infection. Our results suggest that defensins may play a lesser role than LL-37 in host defense against *H*. *ducreyi*.

## Supporting Information

S1 Fig PEA transferases of *H*. *ducreyi* confer resistance to α-defensins HNP-1 and HNP-2 and to β-defensin HBD-2. 35000HP/pLSSK, 35000HPΔPEAT/pLSSK, and 35000HPΔPEAT/pPEAT were tested for resistance to the α-defensins (A) HNP-1 and (B) HNP-2 and (C) the β-defensin HBD-2. Asterisks indicate statistically significant differences from 35000HP (*P* < 0.05). Complementation with pPEAT restored parental levels of susceptibility to defensins. Data represent average ± standard error of three to four independent replicates, and statistical significance was determined by Student’s t-test with Sidak adjustment for multiple comparisons.
(TIF)

S2 Fig Loss of putative PEA transferases *ptdA* and *ptdB* contributes to increased cell surface negativity. The cell surface charges of 35000HP, 35000HP*ΔlptA* (labeled as *ΔlptA*), 35000HP*ΔptdA* (labeled as *ΔptdA*), 35000HP*ΔptdB* (labeled as *ΔptdB*), 35000HP*ΔptdA ptdB* (labeled as *ΔptdA ptdB*), and 35000HPΔPEAT (labeled as ΔPEAT) were examined. The percentage of Alcian blue dye that bound to the bacteria, which correlates with a negatively charged cell surface, was normalized to 35000HP for each sample. Data represent the average ± standard error of five independent assays. An analysis was performed using a linear regression of the number of genes against the raw values, including a random effect for date to account for the correlation among values from the same experiment. A significant trend was observed (P = 0.036).
(TIF)
