# Supplementary figures and images for: Phosphoethanolamine Transferase LptA in Haemophilus ducreyi Modifies Lipid A and Contributes to Human Defensin Resistance In Vitro
Source: PLoS One. 2015 Apr 22;10(4):e0124373. doi: 10.1371/journal.pone.0124373 (PMC4406763; doi:10.1371/journal.pone.0124373)

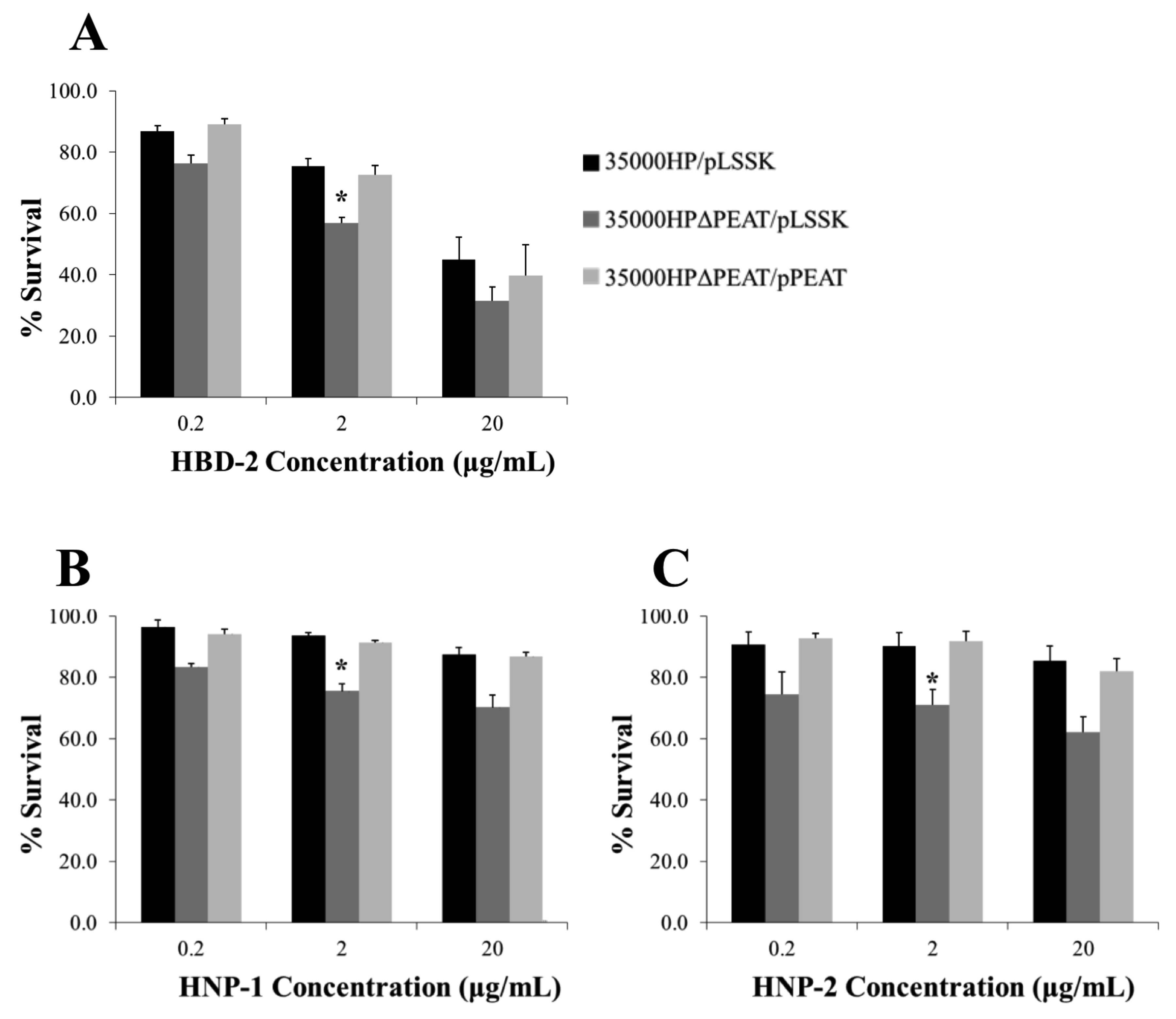

Supplement: S1 Fig — 35000HP/pLSSK, 35000HPΔPEAT/pLSSK, and 35000HPΔPEAT/pPEAT were tested for resistance to the α-defensins (A) HNP-1 and (B) HNP-2 and (C) the β-defensin HBD-2. Asterisks indicate statistically significant differences from 35000HP (P < 0.05). Complementation with pPEAT restored parental levels of susceptibility to defensins. Data represent average ± standard error of three to four independent replicates, and statistical significance was determined by Student’s t-test with Sidak adjustment for multiple comparisons. (TIF) [file pone.0124373.s001.tif]

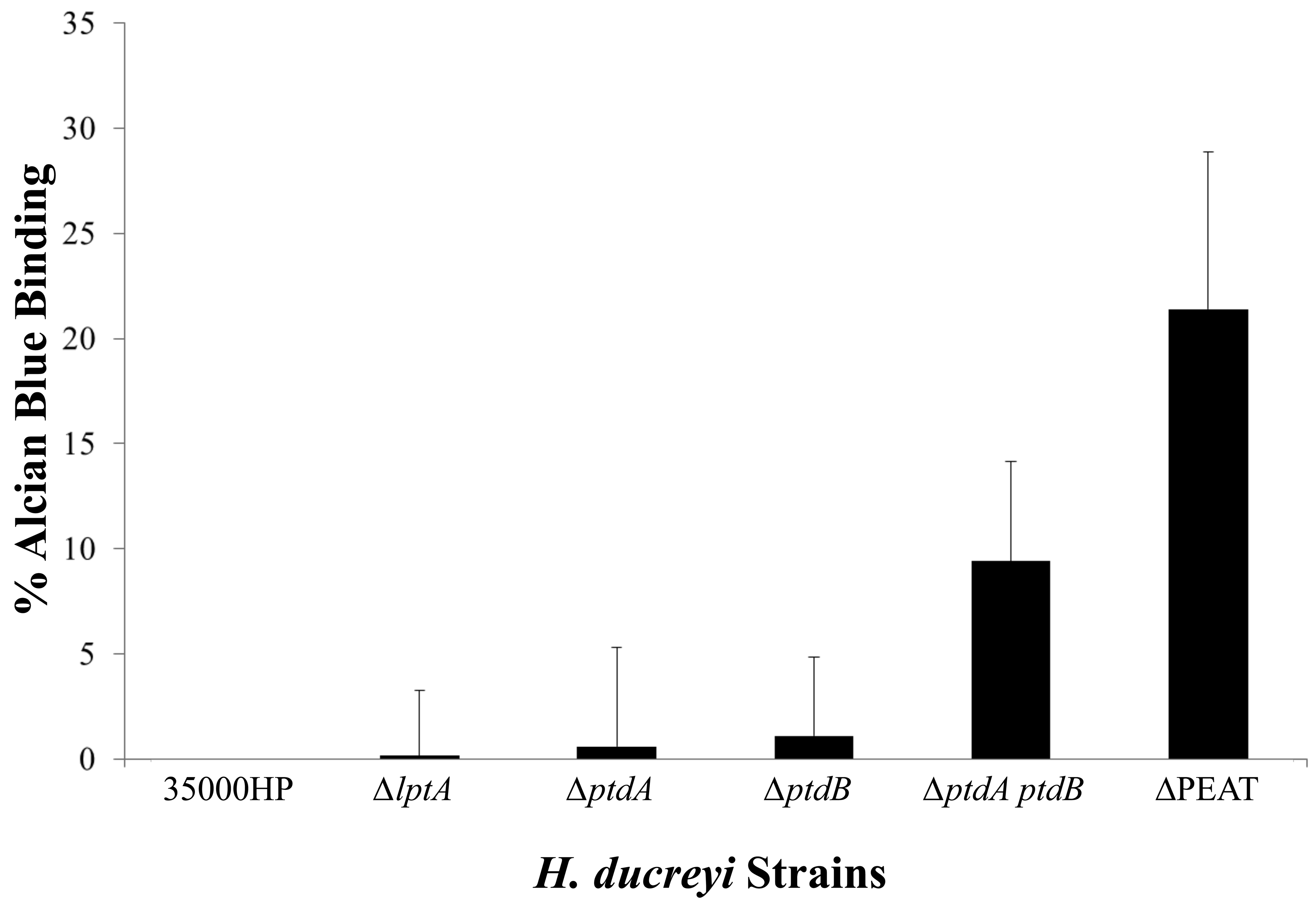

Supplement: S2 Fig — The cell surface charges of 35000HP, 35000HPΔlptA (labeled as ΔlptA), 35000HPΔptdA (labeled as ΔptdA), 35000HPΔptdB (labeled as ΔptdB), 35000HPΔptdA ptdB (labeled as ΔptdA ptdB), and 35000HPΔPEAT (labeled as ΔPEAT) were examined. The percentage of Alcian blue dye that bound to the bacteria, which correlates with a negatively charged cell surface, was normalized to 35000HP for each sample. Data represent the average ± standard error of five independent assays. An analysis was performed using a linear regression of the number of genes against the raw values, including a random effect for date to account for the correlation among values from the same experiment. A significant trend was observed (P = 0.036). (TIF) [file pone.0124373.s002.tif]
